# Association of Aryl Hydrocarbon Receptor-Related Gene Variants with the Severity of Autism Spectrum Disorders

**DOI:** 10.3389/fpsyt.2016.00184

**Published:** 2016-11-16

**Authors:** Takashi X. Fujisawa, Shota Nishitani, Ryoichiro Iwanaga, Junko Matsuzaki, Chisato Kawasaki, Mamoru Tochigi, Tsukasa Sasaki, Nobumasa Kato, Kazuyuki Shinohara

**Affiliations:** ^1^Department of Neurobiology and Behavior, Graduate School of Biomedical Sciences, Nagasaki University, Nagasaki, Japan; ^2^Research Center for Child Mental Development, University of Fukui, Fukui, Japan; ^3^Department of Occupational Therapy, Graduate School of Health Sciences, Nagasaki University, Nagasaki, Japan; ^4^Nagasaki Municipal Welfare Center for the Handicapped, Nagasaki, Japan; ^5^Sasebo Child Development Center, Sasebo, Japan; ^6^Department of Neuropsychiatry, Teikyo University School of Medicine, Tokyo, Japan; ^7^Department of Physical and Health Education, Graduate School of Education, The University of Tokyo, Tokyo, Japan; ^8^Medical Institute of Developmental Disabilities Research, Showa University, Tokyo, Japan

**Keywords:** autism spectrum disorder, aryl hydrocarbon receptor, aryl hydrocarbon receptor nuclear translocator, polymorphism, social communication, severity

## Abstract

Exposure to environmental chemicals, such as dioxin, is known to have adverse effects on the homeostasis of gonadal steroids, thereby potentially altering the sexual differentiation of the brain to express autistic traits. Dioxin-like chemicals act on the aryl hydrocarbon receptor (AhR), polymorphisms, and mutations of *AhR*-related gene may exert pathological influences on sexual differentiation of the brain, causing autistic traits. To ascertain the relationship between *AhR*-related gene polymorphisms and autism susceptibility, we identified genotypes of them in patients and controls and determined whether there are different gene and genotype distributions between both groups. In addition, to clarify the relationships between the polymorphisms and the severity of autism, we compared the two genotypes of *AhR*-related genes (rs2066853, rs2228099) with the severity of autistic symptoms. Although no statistically significant difference was found between autism spectrum disorder (ASD) patients and control individuals for the genotypic distribution of any of the polymorphisms studied herein, a significant difference in the total score of severity was observed in rs2228099 polymorphism, suggesting that the polymorphism modifies the severity of ASD symptoms but not ASD susceptibility. Moreover, we found that a significant difference in the social communication score of severity was observed. These results suggest that the rs2228099 polymorphism is possibly associated with the severity of social communication impairment among the diverse ASD symptoms.

## Introduction

Reports of the incidence of autism spectrum disorder (ASD) – characterized by two core symptoms: communication and social deficits and fixed or repetitive behavior (1) – have been increasing in recent years (2). The prevalence of ASD rose from 1 per 5,000 children in 1975 to 1 per 110 children in 2009 in the United States (3). Current estimates of the break up of possible reasons are as follows: about 25%, attributed to diagnostic accretion; 15%, to the growing awareness of ASD; 10%, to advanced parental age; and 4%, to geographic clustering. However, for the remaining 46% of the cases, the underlying reasons remain unclear (3). Although a strong genetic contribution to ASD has been suggested by many previous studies (4, 5), the syndrome has many features that are not well explained by genetic factors alone (6). Therefore, some researchers have considered projecting ASD as a multifactorial disorder with both genetic and environmental influences (7–9).

The “extreme male brain” (EMB) theory is one of the leading hypotheses for explaining the mechanism of ASD (10, 11). The EMB theory suggests that exposure to imbalanced levels of gonadal steroids (androgen and estrogen) could exert a pathological influence on the sexual differentiation of the brain during the fetal period, which may cause ASD traits in such individuals. Prenatal gonadal steroid levels in the amniotic fluid are correlated with ASD traits in children at 12 and 24 months of age (12, 13). Exposure to environmental chemicals, especially dioxin-like chemicals, is known to have adverse effects on the homeostasis of gonadal steroids, thereby altering the sexual differentiation of the brain to express ASD traits. Dioxin-like chemicals, such as tetrachlorodibenzo-*p*-dioxin (TCDD), polychlorinated dibenzo-*p*-dioxins and dibenzofurans (PCDD/Fs), and some polychlorinated biphenyls (PCBs), act on the aryl hydrocarbon receptor (AhR) (14, 15). Recent accumulating evidence suggests that ligand-activated AhR might alter both estrogen and androgen signals (16, 17). These findings further support that dioxin-like chemical exposure during the fetal period may exert pathological influences on sexual differentiation of the brain, causing ASD traits (18).

Epidemiological studies have shown that PCB exposure at low levels can exert adverse clinical and subclinical effects on sociocognitive functions (19–23). Depending on geographical location, children might be exposed varying background levels of toxic environmental chemicals. Whether the adverse effects are expressed or not depends on inherent individual vulnerability to the environmental chemicals. Therefore, with regard to dioxin-like chemicals in particular, the vulnerability could be modified by an individual’s receptor (*AhR*)-related gene polymorphisms.

Numerous studies have investigated the association between *AhR*-related gene polymorphisms and reproductive system diseases, such as endometriosis or infertility (24–27), because these diseases are regarded as complex traits in which genetic and environmental factors contribute to the disease phenotype (28). Various studies on *AhR*-related gene polymorphisms, as explored by a recent meta-analysis on endometriosis risk in Asian populations (27), have considered *AhR* Arg554Lys and AhR nuclear translocator (*ARNT)* Val189Val as potential candidates (24–27). Although it may be hypothesized that these two polymorphisms contribute to the ASD phenotype by modulating vulnerability to the environmental chemicals, little evidence is available on the relationship between ASD and *AhR*-related gene polymorphisms in humans, with the exception of the investigation on *ARNT2* polymorphisms (29). Therefore, the current study aimed to determine whether polymorphisms of *AhR*-related genes (*AhR* Arg554Lys and *ARNT* Val189Val) contribute to ASD susceptibility and/or severity.

First, to ascertain the relationship between the two *AhR*-related gene polymorphisms and ASD susceptibility, we identified these genotypes in patients and controls and determined whether there are different gene and genotype distributions between the two groups. Second, to clarify the relationships between the polymorphisms and the severity of ASD, we compared the genotypes of *AhR*-related genes with the severity of ASD symptoms using the Childhood Autism Rating Scale (CARS) (30). Finally, we applied factor analysis to the CARS scale and tried to identify several core symptoms, such as social communication, stereotypies, and sensory abnormalities (31). We also tried to evaluate the relationship between the severity of these symptoms and the *AhR*-related gene polymorphisms because the disease severity is not always consistent across symptoms; rather, the relative severity of different symptoms varies among individual cases (32, 33).

## Materials and Methods

### Participants

Ninety-five children and adults with ASD participated in the present study. Participants with ASD were recruited in two different geographical regions, Tokyo and Nagasaki, in Japan. The participants in Tokyo consisted of 68 ASD patients (58 males and 10 females, mean age = 12.43 ± 7.7 years) and were recruited from the outpatient clinics of the Department of Psychiatry in the University of Tokyo Hospital. The participants in Nagasaki consisted of 27 ASD patients (26 male and 1 female patients, mean age = 11.35 ± 3.2 years), and they were recruited from two day-care facilities in Nagasaki prefecture for patients with developmental disorders. In both areas, the diagnoses were made by two or more senior pediatric-psychiatric clinicians through structured interviews and reviews of clinical records according to the DSM-IV criteria (34). Among the 68 patients in Tokyo, 66 were diagnosed with autistic disorder and 2 with Asperger’s disorder. Among the 27 patients in Nagasaki, 17 were diagnosed with autistic disorder and 10 with Asperger’s disorder. Both patient groups excluded individuals with pervasive developmental disorder, not otherwise specified (PDD-NOS). Participants with severe intellectual disability were excluded if they had a full-scale intelligence quotient (FSIQ) <50 on the Wechsler Intelligence Scale for Children (35), Wechsler Adult Intelligence Scale (36), or the Tanaka–Binet Intelligence Scale (a Japanese revised version of the Stanford–Binet Intelligence Scale) (37).

Additionally, 527 adults (332 men and 195 women patients, mean age = 40.9 ± 9.7 years) were recruited as control participants from the nearby community around the University of Tokyo Hospital, without any psychiatric disorder disturbing their work function. The Mini-International Neuropsychiatric Interview (MINI) (38) and other surveys were administered in the recruitment of controls to exclude those who had current or lifetime history of mental disorders. These individuals were recruited from the community through advertisements as well as an online solicitation.

The race/ethnicity of all participants was Japanese. Potential participants were also excluded if they had any history of substance abuse, recent substance use, head trauma with loss of consciousness, significant fetal exposure to alcohol or drugs, perinatal or neonatal complications, and neurological disorders or medical conditions.

The present study was approved by the Ethical Committees of the University of Tokyo Graduate School of Medicine and the Nagasaki University Graduate School of Biomedical Sciences. All participants or parents of the affected individuals provided written informed consent prior to their participation in this study. The experimental protocol was conducted in accordance with the Declaration of Helsinki.

### Assessment of the Severity of ASD

The severity of ASD was assessed on the basis of the Japanese version of the CARS (30). The assessment by CARS was performed at the same time as genomic sampling in this study. The CARS is a behavior-based clinical scale developed by observation and interaction with ASD patients. The scale has been reported to have a high degree of internal consistency, inter-rater and test–retest reliability, high criterion-related validity, and good discriminant validity (39). The severity was rated for 15 items (“Relationship to People,” “Imitation,” “Emotional Response,” “Body Use,” “Object Use,” “Adaptation to Change,” “Visual Response,” “Listening Response,” “Taste, Smell, Touch Response and Use,” “Fear and Nervousness,” “Verbal Communication,” “Non-verbal Communication,” “Activity Level,” “Level and Consistency of Intellectual Response,” and “General Impressions”) on a scale of 1 (normal for child’s age) to 4 (severely abnormal) in units of 0.5. In this study, experienced clinical psychologists rated the subjects based on behavioral observation and parental reports.

### Genotyping

Genomic DNA was extracted from the peripheral blood using the standard phenol–chloroform method for set A and from the oral mucosa of the participants using the QIAamp DNA Micro Kit (Qiagen, Tokyo, Japan) in set B. All participants were genotyped by real-time polymerase chain reaction (PCR) analysis using Roche LightCycler 480 II (Roche Diagnostics, Tokyo, Japan) for the following two single nucleotide polymorphisms (SNPs): *AhR* codon 554 in exon 10 (G/A, Arg to Lys, rs2066853) and *ARNT* codon 189 in exon 7 (G/C, silent mutation, rs2066853) (24–28). Reactions were performed in 5-μl reactions, each containing 5 ng genomic DNA, 2.75 μl HPLC water, 0.25 μl of each TaqMan probe (Applied Biosystems, Foster City, CA, USA), and 2.5 μl TaqMan PCR Master Mix (Applied Biosystems, Foster City, CA, USA). The PCR cycling conditions consisted of a 10-min cycle at 95°C, followed by 60 cycles of 95°C for 30 s and 60°C for 30 s. Five microliters of HPLC water and Mater Mix were used as a negative PCR control in each amplification. Allele calling was performed using LightCycler CW 1.5 software (Roche Diagnostics).

### Data Analysis

Analyses proceeded in four steps. First, the chi-squared test was used to investigate the relationship between each *AhR*-related gene polymorphism and susceptibility to ASD. Next, analysis of variance (ANOVA) was used to compare the severity of ASD among *AhR*-related gene polymorphisms. Third, to assess the severity corresponding to several core behavioral symptoms of ASD, factor analysis with Varimax rotation for CARS was performed, and the factor score was calculated by regression method for each subject. Finally, ANOVA was also used to assess the effects of *AhR*-related gene polymorphisms for each severity of discriminative behavioral symptoms of ASD identified by the prior factor analysis. The chi-squared test, factor analysis, and multinomial logistic regression analysis were performed using IBM SPSS 20.0 for Windows (Statistical Package for the Social Sciences; IBM). The ANOVA was performed using Anovakun software (version 4.8.0.^1^) in the R software space (version 3.2.0. for Windows, R^2^).

## Results

### Genotypes of *AhR*-Related Genes and Susceptibility to and Severity of ASD

The genotype and allele frequencies of *AhR* codon 554 and *ARNT* codon 189 are shown in Table 1. Fourteen samples with *ARNT* codon 189 in healthy participants were excluded from the data because the signal failed to be detected as a result of misamplification. The genotype distributions were in Hardy–Weinberg equilibrium (*p* > 0.05). Comparisons between genotype groups did not demonstrate statistically significant differences with regard to children’s age, sex, or IQ level. To investigate the relationship between each *AhR*-related gene polymorphism and susceptibility to ASD, the chi-squared test was used to evaluate the genotype distribution according to developmental status. No statistically significant association was observed between any of the polymorphisms and susceptibility to ASD.

**Table 1:** **Genotype and allele frequencies of *AhR* and *ARNT* polymorphisms**.

	ASD		Control			ASD		Control	
Genotype	*n*	%	*n*	%	Allele	*n*	%	*n*	%
***AhR* (rs2066853)**									
GG	24	25.3	160	30.4	G	102	53.7	579	54.9
GA	54	56.8	259	49.1	A	88	46.3	475	45.1
AA	17	17.9	108	20.5					
Total	95	100.0	527	100.0		190	100.0	1054	100.0
***ARNT* (rs2228099)**									
GG	39	41.1	189	35.9	G	120	63.2	624	60.8
GC	42	44.2	246	46.7	C	70	36.8	402	39.2
CC	14	14.7	78	14.8					
Total	95	100.0	513^a^	100.0		190	100.0	1026^a^	100.0

*^a^14 samples with ARNT polymorphism from control participants were excluded from the data because the signal failed to be detected as a result of misamplification*.

Next, to investigate the relationship between each *AhR*-related gene polymorphism and the severity of ASD, one-way ANOVA was used for the total CARS score of ASD patients as the dependent variable and the genotype of each *AhR*-related gene polymorphism as the independent variable. There was a statistically significant difference between genotype groups for *ARNT* polymorphism (rs2228099), as determined by ANOVA [*F*(2,92) = 5.69, *p* < 0.01, effect size *f* = 0.352, power = 0.865]. Holm’s sequentially rejective Bonferroni *post hoc* test revealed that the total CARS score of the GG genotype was statistically significantly higher than those of the GC genotypes [*t*(92) = 3.17, *p* < 0.05], whereas there were no statistically significant differences in score between the CC genotype and both genotype groups [GC-CC: *t*(92) = 2.18; GG-CC: *t*(92) = 0.10]. Additionally, there were no statistically significant differences between genotype groups for *AhR* polymorphism (rs2066853) [*F*(2,92) = 0.26]. Taken together, these results suggest that ARNT polymorphism modified the severity of ASD among the *AhR*-related genes examined in the current study.

### Factor Analysis of CARS

To assess the severity according to several core behavioral symptoms of ASD, factor analysis with Varimax rotation was performed for the CARS data. The analysis produced three factors with eigenvalues greater than one. These factors accounted for 55.1% of the common variance. Table 2 shows the factor loadings and the descriptive statistics for each item of the CARS data. The first factor was “social communication,” which consisted of “verbal communication,” “non-verbal communication,” “imitation,” “visual response,” “relating to people,” and “level and consistency,” and assessed the proficiency of social communication and reciprocity. The second factor was “sensory and emotional response,” which consisted of “activity level,” “object use,” “emotional response,” “taste, smell, touch, and response,” “listening response,” and “fear or nervousness,” and assessed abnormalities of sensory and emotional responses. The third factor was “stereotypies,” which consisted of “total impression,” “adaptation to change,” and “body use” and assessed restricted, repetitive patterns of behavior. These three factors well recapitulated the core behavioral symptoms of ASD, as described by the DSM-5, and the results were consistent to those of a similar previous study applying a factor analysis to CARS data (31). Therefore, we used the factor score for each of the three factors in the association analysis between the genotype of *AhR*-related genes and the severity of each of the three behavioral symptoms of ASD.

**Table 2:** **Factor loadings from factor analysis with Varimax rotation^a^, mean, and SD of the 15 items of the CARS**.

CARS Item	Factor			M (SD)
	Social communication	Sensory and emotional response	Stereotypies	
Verbal communication	0.833			2.46 (0.8)
Non-verbal communication	0.563			2.37 (0.7)
Imitation	0.561			2.03 (0.8)
Visual response	0.457	0.456		2.08 (0.8)
Relating to people	0.447		0.446	2.70 (0.7)
Level and consistency of intellectual response	0.419			2.55 (0.7)
Activity level		0.642		2.16 (0.7)
Object use	0.459	0.539		1.98 (0.7)
Emotional response		0.511		2.68 (0.7)
Taste, smell, touch response and use		0.505		1.97 (0.6)
Listening response		0.504		2.12 (0.6)
Fear or nervousness		0.360		2.34 (0.6)
General impressions	0.365		0.732	2.94 (0.6)
Adaptation to change			0.549	2.36 (0.6)
Body use	0.396	0.391	0.430	2.34 (0.5)

*^a^Only factor loadings >0.35 are reported*.

### Genotypes of *AhR*-Related Genes and the Severity of the Core Behavioral Symptoms of ASD

**Figure 1:**
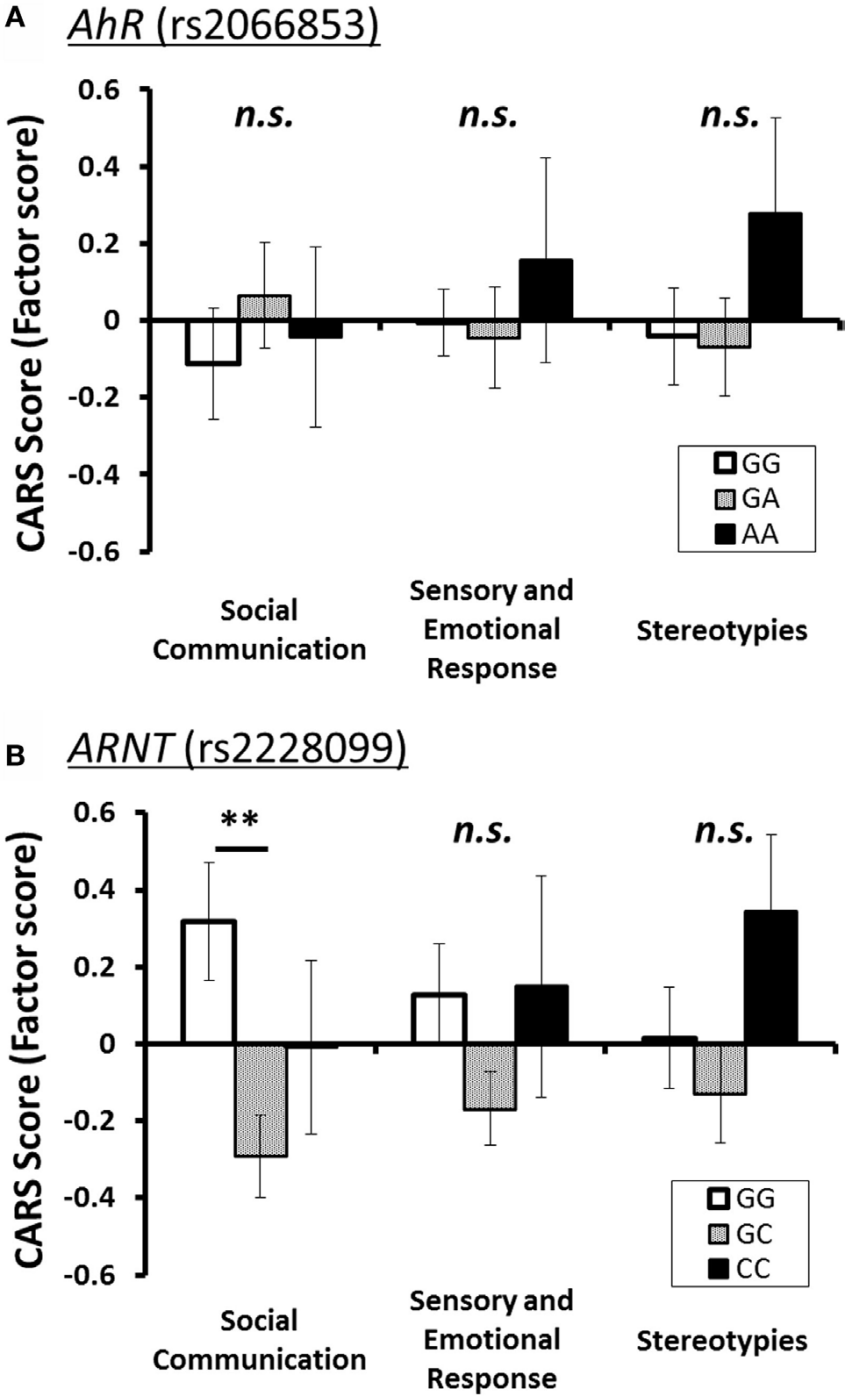
**Three factor scores of CARS (mean ± SE) for each polymorphism of *AhR*-related genes**. The statistical threshold was corrected with Holm’s sequentially rejective Bonferroni adjustment for multiple comparisons. **(A)** *AhR* (rs2066853) and **(B)** ARNT (rs2228099). Note: **p < 0.01.

To investigate relationship between each *AhR*-related gene polymorphism and the severity of several of the core symptoms of ASD, one-way ANOVA was performed for the factor score of CARS of ASD patients as the dependent variable, with the genotype of each *AhR*-related gene polymorphism as the independent variable. Similar to the result for the total score, there were no statistically significant associations between factor scores and genotype groups for *AhR* polymorphism (rs2066853) (Table 3). However, for *ARNT* polymorphism (rs2228099), a significant difference was observed for the factor score of the “social communication” factor, but not for the “sensory and emotional response” factor or “stereotypies” factor. Holm’s sequentially rejective Bonferroni *post hoc* test revealed that the factor score of the GG genotype was statistically significantly higher than that of the GC genotypes [*t*(92) = 3.25, *p* < 0.01], whereas there were no statistically significant differences in score between CC genotype and both genotype groups [GC-CC: *t*(92) = 1.24; GG-CC: *t*(92) = 1.09] (Figure 1).

**Table 3:** **Genotype frequencies of *AhR and ARNT* polymorphisms**.

		Social communication			Sensory and emotional response			Stereotypies		
***AhR* (rs2066853)**										
Genotype		GG	GA	AA	GG	GA	AA	GG	GA	AA
*n*		24	54	17	24	54	17	24	54	17
CARS score	M	−0.11	0.06	−0.04	−0.01	−0.05	0.16	−0.04	−0.07	0.28
	SD	(0.9)	(0.9)	(0.9)	(1.0)	(0.8)	(0.6)	(0.8)	(0.8)	(1.0)
ANOVA	*F*		0.35			0.40			1.15	
	*p*		0.704			0.670			0.320	
Effect size *f*			0.088			0.094			0.158	
Power			0.107			0.116			0.255	
***ARNT* (rs2228099)**										
Genotype		GG	GC	CC	GG	GC	CC	GG	GC	CC
*n*		39	42	14	39	42	14	39	42	14
CARS score	M	0.32	−0.29	−0.01	0.13	−0.17	0.15	0.02	−0.13	0.34
	SD	(1.0)	(0.7)	(0.9)	(0.8)	(0.6)	(1.1)	(0.8)	(0.8)	(0.8)
ANOVA^a^	*F*		**5.29**			1.68			1.72	
	*p*		0.007*			0.191			0.185	
Effect size *f*			**0.339**			0.191			0.193	
Power			**0.838**			0.356			0.363	

^a^The statistical threshold was set at corrected *p < 0.05 (0.05/6 = 0.0083…) with the Bonferroni adjustment for multiple comparisons.

## Discussion

In this study, no statistically significant difference was found between ASD patients and control individuals for the genotypic distribution of any of the polymorphisms studied herein. However, a significant difference in the severity score, especially for the symptom of “social communication,” was observed in *ARNT* codon 189 polymorphism, suggesting that the *ARNT* polymorphism modifies the severity of ASD symptoms but not susceptibility to ASD.

A large twin population study estimated that environmental factors common to twins explain about 55% of the liability to ASD, while genetic factors explain 35% (9). As one of the environmental factors for the liability to ASD, the possible involvement of dioxin and/or dioxin-like environmental chemicals was investigated. Although many environmental chemicals affect neurodevelopment in humans, we focused on dioxin and dioxin-like chemicals because we have shown that higher levels of dioxin-like PCBs in the cord blood appear as a manifestation of ASD-like behaviors in 4-month-old infants (40), and also because maternal exposure to such environmental chemicals possibly disrupts fetal gonadal hormone balances, which could lead to EMB (12, 13). The current study investigated the effects of *AhR*-related gene polymorphisms on ASD susceptibility and/or severity to determine the relationship between possible vulnerability to dioxin and dioxin-like PCBs and ASD susceptibility and/or severity because dioxin and dioxin-like PCBs at low levels have spread almost uniformly throughout the country. *AhR*-related gene polymorphisms have been found to underlie physical diseases such as breast cancer and endometriosis, but no report is available on the effect of these polymorphisms on mental disorders. To the best of our knowledge, the present study is the first report to clarify the association between *ARNT*, an *AhR*-related gene polymorphism, and the severity of ASD symptoms.

The current study could not clarify the mechanism underlying how the *ARNT* polymorphism modifies ASD severity in terms of social communication since no functional analysis of the *ARNT* polymorphism was carried out. However, a possible explanation is that this polymorphism might alter the gonadal hormone balance in the prenatal period through alterations in the AhR signaling pathway and could thus affect ASD severity. It is well known that the sexual differentiation of the human brain depends on prenatal exposure levels of androgens (41), and according to the EMB hypothesis, gonadal hormone imbalances make the autistic brain develop beyond that of the typical male (10, 11). In fact, evidence in favor of the positive association between autistic symptomatology and the levels of fetal testosterone has been found (13, 42). Although there is no evidence for a direct interaction between fetal testosterone and the AhR signaling pathway, the AhR–ARNT heterodimer has been reported to have estrogenic functions in the absence of estrogen (16). Therefore, some sort of functional variant induced by *ARNT* polymorphisms might alter the prenatal exposure levels of gonadal hormone and have adverse effects on sexual differentiation of the brain.

Our preliminary results showed a significant association between the severity of ASD and polymorphism at *ARNT* codon 189, which results in a silent mutation (Val189Val). Although the exact molecular and physiological mechanisms underlying this effect remain unknown, a recent study suggested that silent mutations may contribute to mental disorders (43). Therefore, to clarify the possible meaning of this association, further genetic analyses are necessary. Such analyses should particularly address the interaction with other genetic polymorphisms, both upstream and downstream from rs2228099, which may interfere with splicing and/or *ARNT* mRNA stability.

One of the most interesting findings in the current study is that the *ARNT* polymorphism is specifically associated with the severity of social communication impairment among the diverse ASD symptoms. The diversity of ASD symptoms is an obstacle for elucidating the pathology and etiology, and the core behavioral symptoms defining ASD are genetically heterogeneous in that there are no overlapping genes acting on any of these traits. New, efficient models have been proposed to describe the diverse symptoms by evaluating the severity of the major components of impairments (44, 45). According to this strategy, we surmised that the *ARNT* polymorphism correlated with the severity of social communication difficulties but not with rigid and repetitive behaviors.

Several limitations of the present study should be noted and taken into consideration in future studies. First, the main limitation is the relatively small patient group. We did not observe a significant association between genetic SNPs and susceptibility to ASD in this study, and one possible explanation is the small sample number, as a large number of subjects are needed for case–control studies based on the frequency distribution. Therefore, studies involving a larger number of subjects are essential to generalize our results. Second, the number and age of subjects between the cases and control groups were not well matched. It is possible that the risk modulation by *AhR*-related gene polymorphism depends on the fetal environment and exposure of the mother, which would be expected to vary across different time periods. The disease risk imparted by the allele would therefore be expected to vary in different age groups. Third, although we have used the MINI to ensure the quality of the controls, it is important to note that the MINI does not necessarily exclude ASD. In addition, although the controls also need to be technically screened for family history of ASD or developmental disorders, MINI does not exclude individuals with unidentified Asperger’s or broader autism phenotypes, which is a key concern in the recruitment of controls in this study. In this regard, tools such as the Social Responsiveness Scale (SRS) may have provided a better index (46). This heterogeneity of our sample may be another possible explanation for our negative findings between groups described above. Finally, we used only one clinical scale (CARS) to assess the severity of ASD because we did not obtain full data for any other clinical scale. The ratings by CARS are not invariant across the life span (e.g., non-verbal ability in a 4-year-old individual may not be as severe as that in a 20-year-old individual), although the majority of our clinical samples consisted of children aged 18 years and younger, and the genotype groups were confirmed to not be different with regard to age. However, the heterogeneity in age within our ASD samples could introduce a bias for severity assessment with respect to genetics, and it would also the affect factor analysis process. An informative measure such as SRS adjusted for age may have thus been more useful as a severity measure for purposes of this study (46). Therefore, future studies are needed to assess various aspects of behavioral symptoms of broad social communication using other established scales and to clarify the contribution of *ARNT* gene polymorphism to aspects of social communication.

## Conclusion

In conclusion, the current results showed that individuals with the *ARNT* GG genotype had more severely impaired social communication than those with GC genotype in ASD, indicating that the differences in social functioning in ASD patients may be modulated by *ARNT* variants. Considering that ARNT is a component of AhR cascades, vulnerability to environment chemicals, especially dioxin-like chemicals may affect the severity of impaired social communication, although the functional analysis of *ARNT* gene polymorphism remains to be performed. To identify the neuronal mechanism underlying this effect, combining the present experimental paradigm with neurophysiological indicators of brain activities is warranted in future studies.

## Author Contributions

TF and SN were involved in conducting the experiment, analyzing and interpreting data, and drafting the article. RI, JM, and CK were involved in recruiting the participants and diagnosing the participants with ASD. MT, TS, and NK were involved in conducting the experiment, analyzing and interpreting data, and revising the article. KS conceived of the study, participated in its design and coordination, and drafted the manuscript. All the authors have read and approved the final manuscript.

## Conflict of Interest Statement

The authors declare that the research was conducted in the absence of any commercial or financial relationships that could be construed as a potential conflict of interest.

## Footnotes

^1^http://riseki.php.xdomain.jp.

^2^https://www.r-project.org/.

## Abbreviations

AhR, aryl hydrocarbon receptor; ARNT, aryl hydrocarbon receptor nuclear translocator; ASD, autism spectrum disorder; CARS, Childhood Autism Rating Scale; DSM, diagnostic and statistical manual of mental disorders; EMB, extreme male brain; IQ, intelligence quotient; PBDE, polybrominated diphenyl ether; PCDD/Fs, polychlorinated dibenzo-p-dioxins and dibenzofurans; PCB, polychlorinated biphenyl; SNP, single nucleotide polymorphisms; TCDD, tetrachlorodibenzo-p-dioxin.
